# Patients' Knowledge of Artemisinin-Based Combination Therapy Treatment and Its Impact on Patient Adherence

**DOI:** 10.1155/2018/7465254

**Published:** 2018-07-18

**Authors:** Agani Afaya, Solomon Mohammed Salia, Peter Adatara, Richard Adongo Afaya, Solomon Suglo, Milipaak Japiong

**Affiliations:** ^1^School of Nursing and Midwifery, University of Health and Allied Health Sciences, Ho, Ghana; ^2^Department of Nursing, Kwame Nkrumah University of Science and Technology, Kumasi, Ghana; ^3^Department of Neuro-Surgery, Tamale Teaching Hospital, Ghana

## Abstract

Despite increased support from government and other stakeholders for malaria control over the past decade, malaria burden remains high in many endemic countries, particularly in Sub-Saharan Africa. This study aimed to assess patients' knowledge of antimalarial treatment (ACT) and its association with patient adherence. A descriptive cross-sectional study design was employed in this study. Data were collected from April to May 2017. Both descriptive and inferential statistics in the form of frequencies, percentages, mean values, standard deviations, and Pearson's chi-square test were generated by use of Microsoft excel spreadsheet and IBM Statistical Package for Social Sciences (SPSS) version 23. The average age of the respondents surveyed for this study was 42.27 ± 11.09. Adherence level to ACT was 47%. The results showed that there was a significant association between respondents' knowledge of the efficacy of antimalarial medication (*p = *0.003), benefits of completing antimalarial treatment course (*p =* 0.001), and consequences of not completing the doses of antimalarial medication prescribed (*p =* 0.002) and adherence to ACT. This study then recommends that improving patients' knowledge regarding the efficacy, benefits of completing ACT, and consequences of not completing ACT treatment may improve the likelihood of patients adhering fully to ACT.

## 1. Introduction

Despite increased support from government and other stakeholders for malaria control over the past decade, malaria burden remains high in most endemic nations, predominantly in Sub-Saharan Africa including Ghana [1]. A key malaria control strategy around the world is the prompt treatment with artemisinin-based combination therapy (ACT) targeted towards those confirmed to have malaria [2, 3].

Globally, an estimated 216 million malaria cases occurred in 2016 (95% confidence interval [CI]: 196–263 million), as compared to 237 million malaria cases in 2010 (95% CI: 218–278 million) and 211 million cases in 2015 (95% CI: 192–257 million). A higher percentage (90%) of all malaria cases reported in 2016 occurred in WHO African Countries [4]. Out of the 91 countries that reported malaria cases in 2016, all the 15 countries from Sub-Saharan Africa and India carried 80% of the worldwide malaria burden [4]. The WHO African Region accounted for 91% of all malaria deaths in 2016, followed by the WHO Southeast Asia Region (6%). All the WHO regions recorded drops in mortality rates in 2016 as compared to 2010, with the exception of the WHO Eastern Mediterranean Region, where mortality rates remained virtually unchanged in the period. Similarly, between 2015 and 2016, the mortality rates stalled in the WHO Sub-Saharan Regions calling for prompt measures to reduce the mortalities [4].

Since 1998 till date, Ghana has been a committed member of the Roll Back Malaria (RBM) initiative of the World Health Organisation, which focuses on the Global Malaria Strategy with attention on Sub-Saharan Africa [5]. Following the RBM initiative, Ghana drew up a ‘Medium Term Strategic Plan for Malaria Control' between 1998 and 2002, which sought to improve activities on malaria coverage by adopting an intersectoral approach involving the private sector and the communities. Ghana also committed itself to the Abuja Declaration on RBM in Africa, which is targeted towards malaria prevention and control [5]. In spite of all these initiatives taken by the country, malaria remains hyperendemic and the single most important cause of morbidity and mortality especially among children under 5 years, pregnant women, and the poor [5]. Aside from the health consequences, malaria places a weighty burden on productivity and hence economic growth. Malaria is estimated to cause the loss of about 10.6% of Disability Adjusted Life Years (DALYs) in Ghana costing an equivalent of up to 6% of Gross Domestic Product (GDP) annually in economic burden. Therefore, the GPRS II identifies malaria control and prevention as one of the key health sector interventions [5].

Artemisinin-based combination therapies have been vital to the recent success of malaria control globally, and protecting the efficacy of the ACT for malaria treatment is a global health priority. Though multidrug resistance, including artemisinin (partial) resistance and partner drug resistance, has been reported in 5 countries of the Greater Mekong Subregion (GMS) [6], none has been documented in Sub-Saharan African region on artemisinin resistance. This therefore calls for the need to properly monitor drug efficacy in order to prevent the spread of malaria artemisinin resistance in Africa. Failure to prevent malaria artemisinin resistance would be a setback in the fight against malaria in Africa, as the ACT is the only effective and widely used antimalarial treatment in most health facilities in Africa and other tropical countries.

Patients' knowledge of the correct intake of the proper dosage and timing is a key element in the success of ACT treatment [7]. Poor patient adherence to drugs has serious consequences. For instance, patients who do not complete their treatment course are most likely to have poor recovery rate and exposure to the development of drug resistance [8]. Deportee et al. and Fogg et al. defined adherent patients as those who reported having taken the treatment as recommended (regarding timing and dosage) with no tablets remaining [9, 10]. Several studies have reported patient adherence to ACT across Africa [9–21], but adherence levels varied from less than 30% for ACT in Kenya [22] up to 100% adherence to ACT in Malawi [23]. A recent study by Afaya et al. reported an adherence level of ACT by patients in the Volta Regional Hospital, Ghana, to be 36.6% [21]. In their study, adherence level was measured by patients reporting of completing the treatment course of antimalarial medication (ACT) by themselves [21]. Nonadherence to ACT was attributed to several factors such as the cost of ACT, forgetfulness to take ACT, dislike for medications [11, 21], not improving patient's condition and discontinued medication [14], and improved patient's condition and discontinued medication [17, 24].

Though several studies have been done on patient adherence to antimalarial medication (ACT) and associated factors to nonadherence [19, 21, 25–27], patients' knowledge of ACT (which is key to treatment compliance in malaria) is limited. Patients' understanding of their conditions and treatment is positively related to adherence [28]. Little is known about patient's knowledge of antimalarial treatment (ACT) and its association with ACT adherence. Therefore, this study aimed to assess patients' knowledge of antimalarial treatment (ACT) and its association with patient adherence.

## 2. Materials and Methods

### 2.1. Study Design

A descriptive cross-sectional study design was employed, using a pretested, reliable, and validated semistructured questionnaire to assess patients' knowledge of antimalarial treatment (ACT) and its association to adherence.

### 2.2. Study Site and Population

The study was conducted in the Volta Regional Hospital (VRH). The VRH is in the Ho Municipality in the Volta Region of Ghana. The VRH has a bed complement of about 320, and it is the main referral center for facilities in the region and beyond. The target population for the study included all patients who were admitted at the Medical and Surgical wards and who had been on malaria medication for at least once for the past one year. The average number of cases in the two wards for a month is about 310 cases.

### 2.3. Sampling and Sample Size

Convenience sampling technique was employed in recruiting respondents for the survey. The convenience sampling was used because of its simplicity in recruiting study subjects and proximity to the researchers. The sample size for the study was determined by using Yamane sample size formula [29]. Using an actual population of 310 cases, a sample size of 175 respondents was agreed upon for two months.

### 2.4. Data Collection

After permission and approval were obtained from the VRH and the University of Health and Allied Sciences Ethics Review Committee, the researchers commenced with data collection.

Data were gathered over a period of 8 weeks, from April to May 31, 2017, between the hours of 8 am and 6 pm each day from Monday to Saturday. Trained research assistants were in charge of recruiting and orienting respondents for the study in the various units of the hospital. Written and verbal consent were sought from respondents before recruitment. On the day of recruitment, respondents were educated on the purpose and benefits of the study. Once patients consented, they received the questionnaire which consisted of patients' demographic data and patients' knowledge of antimalarial treatment (ACT). The respondents were informed that participation was voluntary and they could decide whether to participate or not. It was also explained that the respondents could withdraw without penalty at any time during data collection and this would not have any effect on the care they will receive at the facility. An explanation of the expected length of time (approximately 15 minutes) to complete the study questionnaire was provided. The research assistants were present while the respondents completed each questionnaire. Brief instructions for completing each questionnaire was reviewed with respondents to prevent undue burden on them. Most respondents were assisted to complete the questionnaire if they desired so, but respondents who could neither read nor write were assisted by the research assistant to complete the questionnaire. After completion of the questionnaires, the respondents received a copy of the signed consent form and were appreciated as well for taking time to respond to the study. Once completed, all the instruments were placed in an envelope and sealed. The researchers kept completed instruments in a locked desk drawer for safety and confidentiality.

### 2.5. Instrument

A pretested, validated, and reliable semistructured instrument was used for the survey. The instrument was divided into two sections (A and B). Section A had items that measured the demographic characteristics of the respondents; Section B measured patients' knowledge level on antimalarial treatment (ACT).

### 2.6. Data Analysis

Both descriptive and inferential statistics in the form of frequencies, percentages, mean values, standard deviations, and Pearson's chi-square test were generated by the use of Microsoft excel spreadsheet and IBM Statistical Package for Social Sciences (SPSS) version 23. Cross tables of the frequency and percentages of the distribution were produced with regard to the various demographics. The inferential statistics was to establish the statistical significance of the association. *P* values obtained by comparing patient's knowledge scores and adherence were said to be significant if value was less than 0.05. To determine respondents' knowledge score on the treatment of malaria (ACT), respondents were given a score for each correct response to the following variables: name of ACT prescribed, dosage of ACT recommended, efficacy of antimalarial medication, side effects of antimalarial medication, benefits of completing treatment course, duration for taking antimalarial medication, time interval between each dose of antimalarial medication, and consequences of not completing the doses of antimalarial medication prescribed. Respondents who had each of the knowledge components correct were given a score of 1 mark. The overall score of the knowledge components for each of the respondents was 8 marks. The scoring was computerized to arrive at the knowledge score in a descriptive form regarding percentages, mean, and standard deviation.

### 2.7. Validity and Reliability of Instrument

To ensure validity and reliability of the instrument, the researchers were guided by the objective of the study and the reviewed literature in constructing and structuring of the various items. Two professional pharmacists at the VRH and an academic pharmacist face validated semistructured questionnaire. The questionnaire was pretested in the municipal hospital in Ho with 10 patients. The necessary corrections and reconstruction of the questionnaire were made to ensure that every part of the tool reflected the actual situation on the ground. The questionnaire was further retested to ensure its reliability. Different methods have been used to measure medication adherence. Such methods include electronic monitoring devices such as the Medical Event Monitoring System (MEMS), pill counts, self-report through interviews, and biological assays with each of the methods having both advantages and disadvantages. Thus, there is no clear gold standard that has been formalised for measuring adherence for treatment of acute disease like malaria [18, 19]. The study then employed self-report by patients as the ideal method to measure patients adherence level.

### 2.8. Ethical Consideration

Approval to conduct the study was sought from the Volta Regional Hospital and the Research Ethics Committee (REC) of the University of Health and Allied Sciences provided ethical clearance for this study. The purpose and goals of the study were explained to all the respondents. Written and verbal consent were obtained before each questionnaire was administered to the respondents. Respondents were assured of anonymity and confidentiality.

## 3. Results

### 3.1. Demographic Characteristics of Respondents

The average age of respondents surveyed for this study was 42.27± 11.09. Majority of respondents, 107 (representing 62.2%), were with ages ranging from 26 to 45 years. Eighty-nine respondents were females representing 51.7% of the study population. About respondents' religion, the majority (132) were Christians representing 76.7%. With regard to respondents' level of education, the majority (51.2%) of respondents had attained tertiary education. Most respondents (88.4%) reported getting malaria infection about 1-4 times in a year. Also, 149 respondents representing 86.6% of the study population had attended hospital about 1-4 times, and the remaining 23 (13.4%) of the respondents visited the hospital about 5-8 times in a year due to malaria infection. Most respondents (68) representing 39.5% and 54 (31.4%) had their malaria treatment through a doctor's prescription and from licensed chemical sellers, respectively. Most respondents, 90 (52.3%), preferred the oral route of medication for treating malaria. See Table 1 for details on the demographic information of the respondents.

### 3.2. Correlation between Demographic Characteristics and Patients Adherence Level

From Table 2, it was found from the study that age was statistically related to patients' adherence to antimalarial medication with a* P* value of 0.001. Educational status was also significantly related to patients' adherence to antimalarial medication with* P* value of 0.003. Patients' usual malaria treatment and frequency of hospital attendance due to malaria were statistically significant with* P* values of 0.003 and 0.004, respectively. With the overall adherence level, the minority of, 80 (46.5%), respondents were adherent to antimalarial medications.

### 3.3. Knowledge of Treatment of Malaria (ACT) and Its Relationship to Adherence

From Table 3, about 166 (75%) respondents of the 172 respondents knew the type of ACT prescribed for them to treat malaria. The majority, 169 (98%), of respondents knew the benefits of completing antimalarial medication while 150 (87.2%) out of the 169 (98%) respondents were adherent to antimalarial medication. Also, the minority of, 80 (46%), respondents knew the consequences of not completing the dose of antimalarial prescribed. Out of the minority of respondents, 80 (46%), that had knowledge of the consequences of not completing antimalarial medication, 78 (97.5%) were adherent to antimalarial medication. Respondents' knowledge of the efficacy of antimalarial medication was statistically significant with* p* value of 0.003. Also, respondents' knowledge of the benefits of completing antimalarial treatment course was highly significant with* p* value of 0.001. Respondents' knowledge of the consequences of not completing the doses of antimalarial prescribed was statistically related to* p* value of 0.002. The overall knowledge score of the antimalarial treatment of respondent was 79% out of 100%, and respondents' knowledge of the “benefits of completing treatment course” was the highly rated knowledge score of about 98%. Also, the mean for patients' knowledge score on malaria treatment was 6.282 (SD=1.718).

### 3.4. Bivariate and Multivariable Logistic Regression Analysis of Patient Knowledge of Antimalarial Treatment (ACT) with Adherence

Table 4 shows that patients that had knowledge of efficacy of antimalarial treatment (ACT) [AOR = 4.22; 95% CI (1.7, 15.01)] and the benefits of completing treatment course [AOR = 4.10; 95% CI (0.34, 13.0)] had 4 times higher odds of adhering to ACT than those that did not have knowledge of antimalarial treatment (ACT). Similarly, patients that knew the consequences of not completing the doses of prescribed antimalarial medication had 6 times higher odds of adhering to ACT than patients that did not have knowledge of ACT.

## 4. Discussion

This study assessed respondents' knowledge of antimalarial treatment (ACT) and its correlation with adherence. Adherence in this study was measured by patients' self-report of completing their ACT treatment, particularly with dosing and timing. The study found that age (*p*< 0.001) was statistically related to patients' adherence to antimalarial medication. The current study's finding is consistent with other previous studies that reported a significant association between age of the respondents and the adherence level [11, 13, 16, 25] to ACT. However, the findings of this study contradict the findings of Amponsah et al. who reported that the age of patients is not statistically associated with the level of adherence to ACT in Ghana [19]. There are also numerous studies that are not congruent with the current study finding. Such studies found age not to be significantly or consistently associated with adherence [9, 10, 14, 24, 30]. The current study further revealed that as age increases more likely respondents would be nonadherent to ACT. Similarly, a study in Mandalay region on AL adherence shows a high adherence level in the youngest age group as compared with the eldest age group [31]. The disparities in the present study finding and the other studies might be due to the varied methodological approaches employed per study conducted. Also, some of the studies were conducted using children to assess their adherence level [24, 30]. One can therefore stipulate that whereas children can be monitored by their parents to ensure strict adherence to antimalarial medication, contributing to higher adherence level, adults might not strictly adhere to treatment due to schedules and other related factors [21]. The current study found the educational status of respondents to be statistically related to patients' adherence to ACT with* p* value of 0.003. Similarly, educational status and literacy were both found to have a statistical relation with ACT adherence in five studies, with higher levels of education and/or literacy positively associated with adherence [9, 10, 13, 16, 19, 25, 30]. Also, in Uganda patients who had attended at least Senior High School were 22% more likely to be adherent (p = 0.024) to malaria treatment [32]. Another study in Kenya found that higher educational status and the ability to read were both significantly associated with adherence to ACT [16]. It is not surprising that educational level was statistically associated with adherence to ACT as many other studies stated above agree with the current finding. An increase in the level of education would influence the health-seeking behaviour of the individual, and it will also enhance patients ability to search the Internet sources or read health-related magazines to know more about his/her condition and the kind of treatment he/she is currently consuming.

There was also a significant association (*p=*0.004) of patients frequent hospital attendance due to malaria with adherence. It was established that the higher the amount of hospital attendance is due to malaria ailment, the more likely the patient would develop the habit of adhering to antimalarial medication (ACT). It was not therefore surprising that patients' “usual malarial treatment” was statistically associated with adherence. The more the patient attended hospital, the more the patient will be exposed to similar malaria medication prescription and will get an adequate education on malaria treatment, probably dosing and timing of taking the drug from the prescriber.

The study also revealed that gender, religion, episodes of malaria, the source of malaria treatment, and preferred treatment route were not statistically associated with patients' adherence to ACT. Christians dominated the study with most of them believing in prayers instead of medication in their successful recovery from malaria. This current study finding coincides with Kemppainen et al. that highlighted the important role that spiritual beliefs and practices play in understanding disease in the African culture and the belief that God is ultimately in control of an individual's physical and spiritual health [33]. Although peoples' beliefs cannot be altered easily, there is the need to consider the belief of patients when offering care or prescribing medication for patients, as this can affect their adherence to the ACT. Some respondents believe that “prescribers place too much trust in medicines” without taking into consideration the spiritual aspect of the disease. This belief could be addressed if clinicians would involve patient when prescribing, where the patient's opinions and beliefs are considered when care is offered.

The mean knowledge score on malaria treatment was 6.282 (SD=1.718), which showed a high knowledge level within a range of 1-8. This high knowledge score can probably be due to the high level of health education on malaria prevention and control in the country [19]. About 90% of respondents were of the opinion that the ACT were effective and also knew the name of the ACT which they have been taking. It was also interesting to note that the majority (98%) of respondents had knowledge of the benefits of completing antimalarial medication and out of the 98%, 87% of the respondents were adherent to antimalarial medication. However, there were some knowledge gaps among respondents. The majority (54%) of respondents did not have knowledge of the consequences of not completing the dose of antimalarial medication prescribed. There was a significant relation between patient knowledge of the efficacy of ACT and adherence. Patients' knowledge of the benefits of completing malaria treatment course was also significantly associated with adherence. The study also revealed that respondents that had adequate knowledge of the efficacy of ACT and the benefits of ACT were four times more likely to adhere to ACT than respondents who did not have adequate knowledge of ACT. There was a significant association between patients' knowledge of the consequences of not completing malaria treatment and adherence. Respondents in this study that had adequate knowledge of the consequences of not completing their malarial treatment were 6 times more likely to adhere to ACT than respondents that did not have adequate knowledge of the consequences of not completing their antimalarial treatment (ACT). Our study finding concurs with Amponsah et al. where they found a significant relationship between patients' knowledge of the consequences of not completing the doses of antimalarial medication dispensed and adherence [19]. This underlines the need for all health workers providing malaria care to teach patients about the dosage, effectiveness, and side effects of the ACT when they are prescribed. Education by nurses on the treatment of malaria could be offered at the point of care with educational materials. For patients who do not have formal education, pictograms could be used to ensure they understand the dosing regimen and consequences of not completing their medicines. Also, doctors at the point of prescribing could educate patients on the efficacy, dosing, and side effects of the medication. The more the patients would have knowledge of ACT treatment, the more likely the patient would adhere to the treatment regimen. Our study findings are similar to those of Lawford et al. in which they highlighted that improving patient knowledge regarding the Artemether-Lumefantrine dosing regimen may improve the likelihood of patients adhering fully to Artemether-Lumefantrine [11].

The overall patient knowledge of antimalarial treatment (ACT) was 79%. It is quite surprising to note that, despite 79% of respondents having knowledge of antimalarial treatment, respondents did not have a high level of adherence to antimalarial medication (ACT). An adherence level of 47% was low as compared to respondents' high knowledge level of antimalarial treatment. Adherence level was measured by respondents self-reporting of completing the treatment course of antimalarial medication. The study findings revealed an adherence level of 47% which is consistent with Depoortere et al. where they found an adherence level as low as 39.4% in a Zambian refugee settlement [9]. Ngasala et al. findings on adherence were as low as 37% in Tanzania using blood Lumefantrine levels [34], a little low as compared to the current findings, and Lemma et al. found similar results (38.7%) using self-report and pill counts [14]. The current study finding disagrees with Aung et al. where 85.7% of respondents in Rakhine State, Myanmar, were classified as probably adherent and (14.3%) as a nonadherent group. Adherence levels measured on the upper end of the spectrum was as high as 100% in Malawi [23], 98.5% in Uganda [24], and 89.5% in Myanmar [35], all using self-report and pill counts. Also, a study by Kabanywanyi et al. on adherence to and acceptability of Artemether-Lumefantrine as a first-line antimalarial treatment in a Tanzanian community revealed an adherence level of 89.2% which is also inconsistent with the present study findings [12]. These disparities with the current study finding and the other studies might be due to the varied methodological approach used per study conducted. Also, some of the studies were conducted using children to assess their adherence level; one might stipulate that children can be monitored by their parents for strict adherence to antimalarial medication, thereby resulting in higher adherence level. However, in adults as a result of their busy schedules, most people do not adhere to antimalarial medication due to varied reasons or factors.

The lack of homogeneity in the results of the current study and the several other studies especially the large range in adherence levels of ACT can be attributed not only to the differences between the study settings, study designs, and ACT formulations, but also the differences in study implementation such as the study questionnaire and blinding participants/patients to follow-up [18]. For example, the study questionnaire used by Kabanywanyi et al. in their study in Kenya to assess AL adherence was semistructured with open-ended questions embedded within the questionnaire [12], whereas the questionnaire used by Lawford et al. to assess AL adherence in Kenya was more structured and resembled a malaria indicator survey and thus collected a different type of data [11].

## 5. Limitation

Since adherence to artemisinin-based combination therapy was based on the patients' self-reports, though proved reliable, there was the possibility that respondents would have the difficulty to recall whether dosing and timing was followed as recommended by the prescriber.

## 6. Conclusion

The study found a high level of knowledge among respondents. However, the overall high knowledge (79%) of ACT treatment did not reflect patients' antimalarial medication adherence as the study revealed adherence level below average (47%). The current study found a significant association between the following knowledge components: the efficacy of malaria treatment, benefits of completing treatment course and consequences of not completing the doses of antimalarial prescribed, and adherence. This study based on the findings recommends improvement in patients' knowledge regarding the efficacy and the benefits of completing treatment course as well as the consequences of not completing ACT treatment. These may improve the likelihood of patients adhering to ACT treatment.

## Figures and Tables

**Table 1:** Demographic characteristics of respondents.

**PARAMETER**	**FREQUENCY**	**PERCENTAGE (**%**)**
**Total**	**172**	**100**
**Age Category**		
18-25yrs	13	7.6
26-45yrs	107	62.2
36-49yrs	36	20.9
50 years & above	16	9.3
Minimum=18, Maximum=63	Mean=42.27	SD=11.09
**Gender**		
Male	83	48.3
Female	89	51.7
**Religion**		
Traditional	11	6.4
Christian	132	76.7
Muslim	23	13.4
Other	6	3.5
**Level of Education**		
None	6	3.5
Basic	36	20.9
Secondary	42	24.4
Tertiary	80.8	51.2
**Episodes of Malaria in last year**		
1-4 times	152	88.4
5-8 times	20	11.6
**Freq. of hospital attendance for malaria**		
0-4 times	149	86.6
5-8 times	23	13.4
**Source of malaria treatment**		
Herbal preparation at home	50	29.1
Self-Medication from chemical shops	54	31.4
Doctor's prescription	68	39.5
**Preferred Treatment route**		
Oral tablet	90	52.3
Injectable	16	9.3
Both oral and injectable	66	38.4

**Table 2:** Respondents' adherence stratified by the demographic characteristics.

**DEMOGRAPHIC CHARACTERISTICS**		**ADHERENCE**		**TOTAL**	**Pearson's Chi-Square Test**
		**Adherent**	**Non-Adherent**		***p*-Value**
Gender	Male	41(49.4)	42(50.6)	83(100)	0.281
	Female	39(43.8)	50(56.2)	89(100)	

Age	18-25 yrs.	5(38.5)	8(61.5)	13(100)	0.001*∗*
	26-40 yrs.	57(53.3)	50(46.7)	107(100)	
	41-55 yrs.	16(44.4)	20(55.6)	36(100)	
	55 and above	2(12.5)	14(87.5)	16(100)	

Religion	Traditional	6(54.5)	5(45.5)	11(100)	0.002
	Christian	67(50.8)	65(49.2)	132(100)	
	Muslim	4(17.4)	19(82.6)	23(100)	
	Other	3(50.0)	3(50.0)	6(100)	

Education	None	0(0.0)	6(100.0)	6(100)	0.003^*∗*^
	Secondary	13(31.0)	29(69.0)	42(100)	
	Basic	14(39.0)	22(61.0)	36(100)	
	Tertiary	53(60.2)	35(39.8)	88(100)	

Episodes of Malaria	1-4 times	75(49.3)	77(50.7)	152(100)	0.063
	5-8 times	5(25.0)	15(75.0)	20(100)	

Freq. of hospital attendance due to malaria	0-4 times	74(49.7)	75(50.3)	149(100)	0.004*∗*
	5-8 times	6(26.0)	17(74.0)	23(100)	

Usual malaria treatments	ACTs	51(53.0)	45(47.0)	96(100)	0.003*∗*
	Herbal Drug	23(45.0)	28(55.0)	51(100)	
	Both herbal drug & ACTs	6(24.0)	19(76.0)	25(100)	

Source of treatment	Herbal preparation at home	22(44.0)	28(56.0)	50(100)	0.175
	Self-Medication from shop	21(39.0)	33(61.0)	54(100)	
	Doctor's prescription	37(54.4)	31(45.6)	68(100)	

Preferred Treatment route	Oral tablet	32(35.6)	58(64.4)	90(100)	0.058
	Injectable	7(43.8)	9(56.3)	16(100)	
	Both oral and injectable	41(62.0)	25(38.0)	66(100)	

**TOTAL**		**80(47)**	**92(54)**	**172(100)**	

P<0.005=statistically significant^*∗*^ .

**Table 3:** Knowledge of treatment of malaria and its relationship to adherence.

**Respondents awareness**	**N (**%**)**	**Adherent**	**Non-Adherent**	***P* Value**
Name of ACTs prescribed	166(96)	101	65	0.056
Dosage of ACTs recommended	135(78)	79	56	0.089
Efficacy of antimalarial medication	153(90)	140	13	0.003^*∗*^
Side effects of antimalarial medication	100(58)	45	55	0.191
Benefits of completing treatment course.	169(98)	150	19	0.001^*∗*^
Duration for taking antimalarial medication.	127(74)	100	27	0.293
Time interval between the next doses of antimalarial medication.	100(58)	78	22	0.053
Consequences of not completing the doses of antimalarial medication prescribed	80(46)	78	2	0.002^*∗*^

**Overall knowledge score**	**79**%			

**Table 4:** Bivariate and multivariable logistic regression analysis of patient knowledge on antimalarial treatment (ACTs) with adherence.

**Respondents awareness**	**K Level**	**Not Adh **	**Adh**	**Total N(**%**)**	**COR [95**%**CI]**	**AOR [95**%**CI]**
Efficacy of malarial treatment	Kn	7	120	127(83)	5.14[2.6, 12.52]	4.22[1.7, 15.01]
	Not Kn	6	20	26(17)	1.0	1.0

Benefits of completing treatment course.	Kn	12	140	152(90)	6.36[1.3, 14.9]	4.10[0.34, 13.0]
	Not Kn	6	11	17(10)	1.0	1.0

Consequences of not completing the doses of antimalarial medication prescribed	Kn	3	77	80 (46)	7.13[3.4, 20.7]	6.05[1.29, 28.3]
	Not Kn	20	72	92(54)	1.0	1.0

Kn: knowledgeable.

## Data Availability

The data used to support the findings of this study are available from the corresponding author upon request.

## References

[1] World Health Organization WHO Global Malaria programme. World Malaria Report 2014. http://www.rollbackmalaria.org/files/files/about/9789241564830_eng.pdf.

[2] WHO T3: Test. Treat. Track Initiative.

[3] WHO (2010). *Guidelines for the Treatment of Malaria*.

[4] WHO World Malaria Report 2017, Geneva. https://www.reliefweb.int/sites/reliefweb.int/files/resources/WMR-2017-slide-deck-briefings.pdf.

[5] Ministry of Health (MOH) Anti-Malaria Drug Policy for Ghana. http://www.apps.who.int/medicinedocs/en/d/Js18072en/.

[6] WHO Artemisinin and artemisinin-based combination therapy resistance. Global Malaria Programme. https://www.apps.who.int/iris/bitstream/10665/255213/1/WHO-HTM-GMP-2017.9-eng.pdf.

[7] Adjuik M., Babiker A., Garner P. (2004). Artesunate combinations for treatment of malaria: meta-analysis. *The Lancet*.

[8] White N. J., Olliaro P. L. (1996). Strategies for the prevention of antimalarial drug resistance: rationale for combination chemotherapy for malaria. *Parasitology Today*.

[9] Depoortere E., Guthmann J.-P., Sipilanyambe N. (2004). Adherence to the combination of sulphadoxine-pyrimethamine and artesunate in the Maheba refugee settlement, Zambia. *Tropical Medicine & International Health*.

[10] Fogg C., Bajunirwe F., Piola P. (2004). Adherence to a six-dose regimen of artemether-lumefantrine for treatment of uncomplicated Plasmodium falciparum malaria in Uganda. *The American Journal of Tropical Medicine and Hygiene*.

[11] Lawford H., Zurovac D., O'Reilly L. (2011). Adherence to prescribed artemisinin-based combination therapy in Garissa and Bunyala districts, Kenya. *Malaria Journal*.

[12] Kabanywanyi A. M., Lengeler C., Kasim P. (2010). Adherence to and acceptability of artemether-lumefantrine as first-line anti-malarial treatment: evidence from a rural community in Tanzania. *Malaria Journal*.

[13] MacE K. E., Mwandama D., Jafali J. (2011). Adherence to treatment with artemether-lumefantrine for uncomplicated Malaria in Rural Malawi. *Clinical Infectious Diseases*.

[14] Lemma H., Löfgren C., Sebastian M. S. (2011). Adherence to a six-dose regimen of artemether-lumefantrine among uncomplicated *Plasmodium falciparum* patients in the Tigray Region, Ethiopia. *Malaria Journal*.

[15] Simba D. O., Kakoko D., Tomson G. (2012). Adherence to artemether/lumefantrine treatment in children under real-life situations in rural Tanzania. *Transactions of the Royal Society of Tropical Medicine and Hygiene*.

[16] Onyango E. O., Ayodo G., Watsierah C. A. (2012). Factors associated with non-adherence to Artemisinin-based combination therapy (ACT) to malaria in a rural population from holoendemic region of western Kenya. *BMC Infectious Diseases*.

[17] Ogolla J. O., Ayaya S. O., Otieno C. A. (2013). Levels of adherence to coartem in the routine treatment of uncomplicated malaria in children aged below five years, in Kenya. *Iranian Journal of Public Health*.

[18] Banek K., Lalani M., Staedke S. G., Chandramohan D. (2014). Adherence to artemisinin-based combination therapy for the treatment of malaria: a systematic review of the evidence. *Malaria Journal*.

[19] Amponsah A. O., Vosper H., Marfo A. F. A. (2015). Patient related factors affecting adherence to antimalarial medication in an urban estate in Ghana. *Malaria Research and Treatment*.

[20] Gore-Langton G. R., Alenwi N., Mungai J. (2015). Patient adherence to prescribed artemisinin-based combination therapy in Garissa County, Kenya, after three years of health care in a conflict setting. *Malaria Journal*.

[21] Afaya A., Salia S. M., Opare F. Y., Ali S., Afaya R. A. (2017). Patients’ adherence to antimalarial medication; self-report of patients at the Volta regional hospital of Ho, Ghana. *International Journal of Research in Medical Sciences*.

[22] Watsierah C. A., Jura W. G., Raballah E., Kaseje D., Abong'O B., Ouma C. (2011). Knowledge and behaviour as determinants of anti-malarial drug use in a peri-urban population from malaria holoendemic region of Western Kenya. *Malaria Journal*.

[23] Bell D. J., Wootton D., Mukaka M. (2009). Measurement of adherence, drug concentrations and the effectiveness of artemether-lumefantrine, chlorproguanil-dapsone or sulphadoxine-pyrimethamine in the treatment of uncomplicated malaria in Malawi. *Malaria Journal*.

[24] Kalyango J. N., Rutebemberwa E., Karamagi C. (2013). High adherence to antimalarials and antibiotics under integrated community case management of illness in children less than five years in Eastern Uganda. *PLoS ONE*.

[25] Aung W., Dondorp A. M., Min M. (2015). Assessment of adherence to three days course of Artemether-lumefantrine treatment in Rakhine state, Myanmar. *JITMM Proceedings*.

[26] Chatio S., Aborigo R., Adongo P. B., Anyorigiya T., Akweongo P., Oduro A. (2015). Adherence and uptake of artemisinin-based combination treatments for uncomplicated malaria: a qualitative study in northern Ghana. *PLoS ONE*.

[27] Schallig H. D., Tinto H., Sawa P. (2017). Randomised controlled trial of two sequential artemisinin-based combination therapy regimens to treat uncomplicated falciparum malaria in African children: a protocol to investigate safety, efficacy and adherence. *BMJ Global Health*.

[28] Atreja A., Bellam N., Levy S. R. (2005). Strategies to enhance patient adherence: making it simple.. *MedGenMed : Medscape general medicine*.

[29] Yamane T. (1967). *Statistics, An Introductory Analysis*.

[30] Beer N., Ali A. S., Rotllant G. (2009). Adherence to artesunate-amodiaquine combination therapy for uncomplicated malaria in children in Zanzibar, Tanzania. *Tropical Medicine & International Health*.

[31] Tun Z. W., Lin Z., Wai K. (2012). Adherence to the recommended regimen of artemether-lumefantrine for treatment of uncomplicated falciparum malaria in Myanmar. *Myanmar Health Sciences Research Journal*.

[32] Cohen J. L., Yavuz E., Morris A., Arkedis J., Sabot O. (2012). Do patients adhere to over-the-counter artemisinin combination therapy for malaria? Evidence from an intervention study in Uganda. *Malaria Journal*.

[33] Kemppainen J., Kim-Godwin Y. S., Reynolds N. R., Spencer V. S. (2008). Beliefs about HIV disease and medication adherence in persons living with HIV/AIDS in rural southeastern North Carolina. *Journal of the Association of Nurses in AIDS Care*.

[34] Ngasala B. E., Malmberg M., Carlsson A. M. (2011). Effectiveness of artemether-lumefantrine provided by community health workers in under-five children with uncomplicated malaria in rural Tanzania: an open label prospective study. *Malaria Journal*.

[35] Zaw-Win T., Zaw L., Khin W. (2012). Adherence to the recommended regimen of artemether-lumefantrine for treatment of uncomplicated falciparum malaria in Myanmar. *Myanmar Health Sciences Research Journal*.

